# Thermal dependence of trap building in predatory antlion larvae (Neuroptera: Myrmeleontidae)

**DOI:** 10.1007/s10164-018-0540-5

**Published:** 2018-02-06

**Authors:** Andrzej Antoł, Wiktoria Rojek, Krzysztof Miler, Marcin Czarnoleski

**Affiliations:** Institute of Environmental Sciences, Jagiellonian University, Gronostajowa 7, 30-387 Kraków, Poland

**Keywords:** *Myrmeleon bore*, Predation, Sit-and-wait predators, Thermal behavior, Thermal performance, Trap size

## Abstract

Trap-building predators remain under strong selection from thermal microenvironments. To address how soil temperature and body size affect trap building, we conducted a laboratory experiment using larvae of the antlion *Myrmeleon bore* at six ecologically relevant temperatures. Larger larvae built larger traps, and warmer soil led to more and larger traps. Body mass did not alter the dependence of trap building on temperature. Our results suggest that the physiological capacity of antlion larvae, which is affected by larval size and body temperature, is the major determinant of trap building. This effect should be considered when assessing interactions between antlions and prey.

## Introduction

Body temperature has profound effects on physiological performance, and so it is not surprising that many ectotherms behaviorally maintain a narrow range of body temperatures to stay close to their performance maxima (Angilletta 2009). However, it has become clear that thermoregulatory behavior involves complex decisions that compromise different components of Darwinian fitness, including physiological capacity and survival (Basson et al. 2017). All else being equal, organisms with different degrees of mobility cannot equally engage in thermoregulatory behaviors. This results in different risks of fluctuations in physiological states and of approaching physiological limits.

Sedentary lifestyles have rarely evolved among terrestrial animals, but peculiar exceptions to this include some antlions (Neuroptera: Myrmeleontidae) with sit-and-wait predatory larvae that form conical pitfall traps in the substrate to capture invertebrates (Scharf and Ovadia 2006; Scharf et al. 2011; Hollis et al. 2015). As antlion larvae live at the mercy of local conditions necessary for effective hunting (dry, sandy areas), they should experience selection for increased thermal tolerance and the capacity to perform over a wide range of temperatures. Unfortunately, thermal dependence of hunting behaviors has been studied in only a limited number of antlion species, such as *Myrmeleon immaculatus* and *Euroleon nostras*, which seem to actively prefer shaded areas (Haub 1942; Green 1955; Klein 1982; Heinrich and Heinrich 1984; Arnett and Gotelli 2001; Klokočovnik et al. 2016). Such shade-preferring species may not have as high thermal tolerance as those that do not avoid open areas and occur in fully sun-exposed locations. Indeed, Rotkopf et al. (2012) demonstrated that the larvae of *Cueta lineosa*, which do not avoid open areas, built larger traps and were more active at extreme temperatures than *Myrmeleon hyalinus* larvae, which prefer shaded areas. Other species that do not avoid open areas were shown to thrive at 40–50 °C (Cain 1987; Marsh 1987), the temperature range often regarded as the upper thermal physiological limit for many ectotherms. Interestingly, the larvae of South African *Myrmeleon obscurus* were found to tolerate a very wide range of temperatures, ceasing pit construction below 10 °C and above 42 °C (Youthed and Moran 1969).

To address the effects of the thermal environment on antlion hunting, we conducted a laboratory experiment on trap building in *Myrmeleon bore* larvae along an ecologically relevant thermal gradient. Although *M. bore* has been used to study antlion feeding biology and trap establishment (Matsura 1986, 1987; Matsura and Takano 1989; Abraham 2003; Matsura et al. 2005), thermal biology has never been studied in this species. We tested two competing hypotheses. Given that the capacity to build traps can directly reflect the dependence of larval behavior and metabolism on temperature, we expected soil temperature to positively affect pitfall trap volume (hypothesis 1). Such an effect was previously found in *Myrmeleon pictifrons* (Kitching 1984), *Myrmeleon immaculatus* (Arnett and Gotelli 2001) and *E. nostras* (Klokočovnik et al. 2016). However, other studies showed contrasting effects of temperature on trap building. For example, in another study on *M. immaculatus*, the trap-building frequency was negatively correlated with temperature (Klein 1982). According to Rotkopf et al. (2012), transient alterations in temperature induced the restructuring of traps in *M. hyalinus* and *C. lineosa*, but the initial trap size did not change consistently along a thermal gradient. We hypothesized that the inconsistency in the thermal dependence of trap building might indicate adjustments in trap-building behaviors to the current ability of larvae to handle prey. Antlion larvae use costly to produce chemical paralytics to immobilize prey (Matsuda et al. 1995; Yoshida et al. 1999), indicating that the production of paralytics and prey handling capacity should decrease at lower body temperatures with decrease of metabolic rate. *M. bore* larvae inhabit open xerothermic areas in the temperate zone of Eurasia and, compared to their tropical counterparts, they seem to commonly interact with ant species that have evolved behaviors aimed at rescuing nest mates entrapped by antlion larvae (Miler et al. 2017). The evolution of such ant behaviors in a temperate climate may be driven by the greater success of rescues in confrontations with antlions facing thermal restrictions on their hunting (Miler et al. 2017). Therefore, we hypothesized that cold *M. bore* larvae would build larger pitfall traps to compensate for their reduced capacity to immobilize prey and prevent rescues. This effect would result in larger pitfall trap volumes at lower temperatures or, at the least, no effect of temperature on trap volume in our experiment (hypothesis 2).

## Materials and methods

### Experimental animals

We collected first-instar *M. bore* larvae in October 2016 near Klucze village, from the Błędowska Desert, Poland. After the animals were transferred to the Institute of Environmental Sciences, Jagiellonian University, Kraków, they were maintained individually without food in plastic cups containing dry sand in a thermal cabinet at 22 °C under a 12-h:12-h light:dark (L:D) photoperiod. Species identity was determined according to Badano and Pantaleoni (2014).

The Błędowska Desert originated in the Middle Ages as a result of the intense mining and deforestation in the area of Olkusz. It is now the largest accumulation of loose sand in Central Europe (aside from beaches) (Węgrzyn and Wietrzyk 2014) and it supports a large *M. bore* population. When antlions are active (May–October), the mean temperature in the Błędowska Desert is 14.4 °C and the minimum and maximum temperatures reach − 3.7 and 32.4 °C, respectively (calculated for 2000–2015 from records provided by the nearby Olewin meteorological station of the Institute of Meteorology and Water Management, National Research Institute, Poland).

### Experimental procedure

In total, we tested 57 animals. Prior to the experiment, each larva was weighed to the nearest 0.001 mg (Mettler Toledo XP26, Greifensee, Switzerland) and placed individually in a plastic container with 110 cm^3^ of fine dry sand. The containers were then placed in three thermal arenas (100 cm × 3 cm) in which the temperature was locally controlled using Peltier heat pumps. Both ends of each arena were equipped with Peltier coolers, which were used to generate two distinct temperatures and a continuum between them. To ensure the ecological relevance of our results, we matched the experimental thermal range with thermal conditions characteristic of the active period of *M. bore* in the Błędowska Desert. Each arena was used to generate one of three gradients (15–20, 25–30, 35–40 °C). Thermochron iButtons (Maxim/Dallas Semiconductor, USA) were placed in each container to measure the sand temperature that was directly experienced by the larvae. Note that the 15–20 °C platform was placed in a thermal room at 15 °C, and the other two platforms were placed in another thermal room at 22 °C. These ambient conditions helped maintain the adequate performance of the Peltier coolers. Both rooms had a maintained 12:12 L:D photoperiod. After 24 h of acclimatization to the treatment conditions, we destroyed traps that were built in the experimental containers and allowed the larvae to rebuild traps over the next 24 h. We measured the depth and diameter of the re-established traps (to the nearest millimeter) using a device consisting of a transparent plastic circular plate with a thin rod penetrating a hole in the center. The plate and the rod were equipped with a scale. The rod moved freely through the hole, and during the measurements, it was positioned perpendicularly to the plate, just above the deepest point of the trap being measured. Assuming that the traps were conical, we used the linear measurements to calculate trap volume (cubic centimeters).

### Statistical analysis

Data were analyzed using R software (R Core Team 2015). A generalized linear model (GLM) with a binomial distribution and logit link function was used to examine thermal effects on trap establishment. Each larva was assigned a value of 1 if a trap was established or a value of 0 if a trap was not established. A multiple regression (MR) was used to examine thermal effects on trap volume and trap depth (only considering established traps). All models considered temperature (mean temperature recorded by each iButton) and body mass as continuous predictors and an interaction between the two variables; body mass and trap size (volume and depth) were log transformed prior to analysis.

## Results

**Fig. 1:**
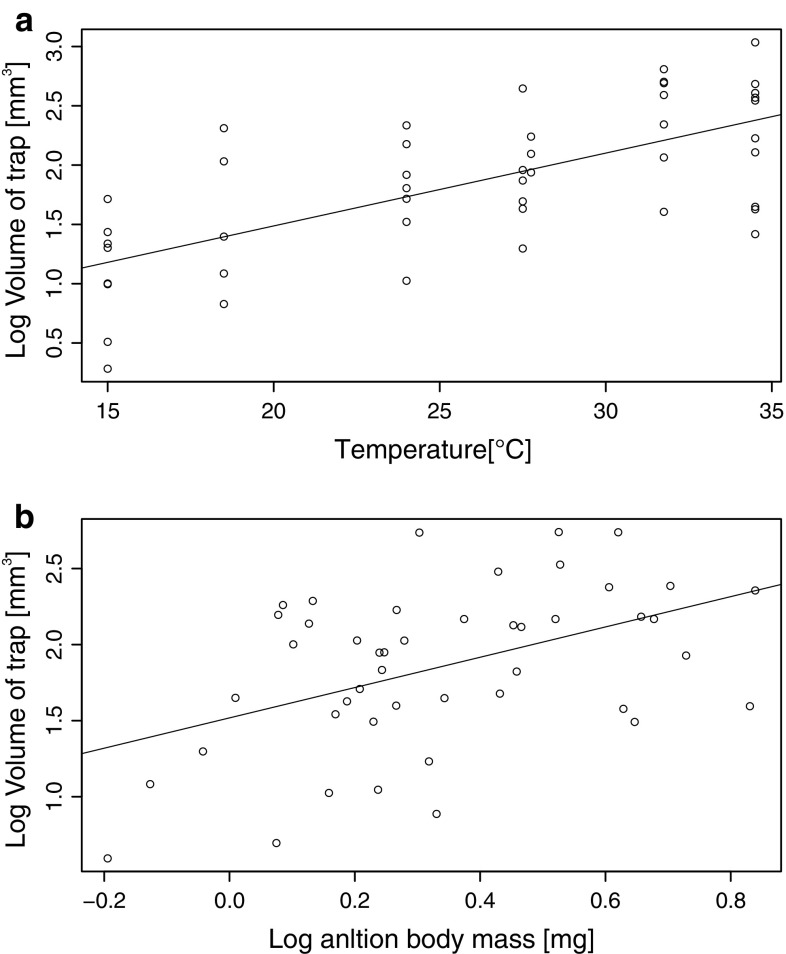
The volume of pitfall traps established by antlion larvae during a 24-h thermal experiment increased with soil temperature (**a**) and larval body mass (**b**). The* lines* fitted to the data represent values predicted by a multiple regression model with temperature and log_10_ body mass as two independent covariates. Each partial regression (**a** *y* = 0.26 + 0.061*x* and **b** *y* = 1.52 + 0.996*x*) was established keeping the other covariate, either log_10_ body mass (**a**) or temperature (**b**), at its mean value

In all statistical models, body mass and temperature did not have a significant interactive effect on the analyzed characteristics of trap building; therefore, we removed this interaction from all final models. The final models showed that more larvae established traps at higher temperatures (GLM, *z*_1,54_ = 1.98, *p* = 0.048) and body mass did not affect the likelihood of trap establishment (GLM, *z*_1,54_ = − 0.21, *p* = 0.83). Higher temperatures also resulted in larger traps (MR, trap volume, *t*_1,43_ = 5.33, *p* < 0.001, Fig. 1a; trap depth, *t*_1,43_ = 5.02, *p* < 0.001), and heavier larvae established larger traps (MR, trap volume, *t*_1,43_ = 3.20, *p* = 0.003, Fig. 1b; trap depth, *t* = 2.69, *p* = 0.01).

## Discussion

The results of our experiment show that higher soil temperatures resulted in more frequent trap building and traps with a larger diameter and volume. These findings are consistent with those from some earlier studies on trap building in antlion larvae (Kitching 1984; Arnett and Gotelli 2001; Klokočovnik et al. 2016), but they contrast with the conclusions of other studies showing either no clear effects of temperature on trap volume (Rotkopf et al. 2012) or negative effects of temperature on trap-building frequency (Klein 1982). Our findings do not support hypothesis 2, which predicts that antlion larvae challenged by cooler conditions might compensate for their reduced behavioral capacity to immobilize prey and prevent rescue by ant nest-mates by maintaining larger traps. Instead, our results support an alternative hypothesis 1, which predicts that trap building is dictated by the thermal dependence of physiological capacity. In addition, building larger cone-shaped traps in response to stronger solar radiation might signify thermoregulatory behavior. Indeed, the base of pitfall traps has been shown to provide larvae with lower mean temperatures and reduced thermal fluctuations compared to the upper surface of the sand, where larvae establish their traps (Green 1955; Marsh 1987; Abraham 2003). In our experiment, we heated a layer of sand from the bottom, which means that by choosing to build a larger trap, a larva was closer to a heat source than a larva building a smaller trap. Thus, we conclude that effects on physiological capacity are the most likely explanation for the positive relationship between temperature and trap building. Note that the role of physiological capacity in trap building is further supported by our finding that the volume of traps was strongly determined by the body mass of larvae (isometric mass-scaling, see Fig. 1b). The positive effect of larval size on trap size was previously reported, e.g., by Heinrich and Heinrich (1984), Kitching (1984) and Youthed and Moran (1969), but we were able to demonstrate this effect over a very narrow range of body masses in relatively small larvae (1–10 mg). According to Heinrich and Heinrich (1984), the positive effects of larval size on trap size can become weaker as larvae grow in size. Interestingly, we found that the thermal dependence of trap building did not change with the body mass of antlion larvae, contrary to the idea that temperature and body size elicit interactive effects on the metabolic performance of ectotherms (Glazier 2005). According to Kozlowski et al. (2004), the nature of thermal and size dependence of physiological performance plays a crucial role in the evolution of thermal plasticity in the life histories of ectotherms.

If trap building reflects the physiological and behavioral performance of antlion larvae, then why have there been conflicting results regarding the thermal dependence of this behavior? One likely explanation is the choice of experimental temperatures combined with the choice of species. In ectotherms, traits related to physiological performance typically display a bell-shaped thermal response: performance increases with temperature, the performance reaches its maximal level at some temperature, and then it dramatically decreases with further temperature increases. Different phenomena, including the nonlinear nature of protein kinetics or the misbalance between supply and demand, have been proposed to shape such a thermal response (reviewed extensively by Angilletta 2009). It is likely that studies demonstrating a positive effect of temperature on trap building focused on a lower range of temperatures than studies demonstrating a negative effect. Furthermore, given that optimal body temperatures might differ between species that prefer shaded areas and species that are unresponsive to shade (see “Introduction”), the same thermal range could result in contrasting thermal responses between such species. Some evidence supports this view. For example, Green (1955) showed that the intensity of hunting behaviors in *M. immaculatus* larvae (mandible extensions, body movements or direct prey capture attempts) increased with temperature to some point, but decreased with further increases in temperature. Klein (1982) concluded that antlion larvae of the same species increased their trap-building frequency at lower temperatures, but Green’s (1955) results suggest that Klein used a thermal range (38–47 °C) that may exceed the thermal tolerance limit of this species. Interestingly, Katz et al. (2017), demonstrated that the larvae of wormlions, another group of insects (Diptera: Vermileonidae) that, independently of antlions, evolved sit-and-wait predatory larvae that build pitfall traps, built the largest traps at a moderate temperature when exposed to three different thermal treatments, supporting the idea of a bell-shaped thermal dependence of trap building. However, prior acclimation of wormlions to thermal treatments decreased the thermal sensitivity of trap building.

Overall, our findings have important implications for further research in the field of antlion larvae ecology. Given that larger traps increase the access of antlion larvae to larger prey (Heinrich and Heinrich 1984; Mansell 1988; Devetak 2005; Humeau et al. 2015), the relative predation pressure of antlion larvae on large vs. small invertebrates seems to change with the size and thermal environment of larvae. Given our results on the dependence of trap building on size, as an antlion larva grows in size, which can take up to 3 years in temperate regions (Scharf and Ovadia 2006; Scharf et al. 2011; Hollis et al. 2015), its capacity to prey on larger invertebrates increases systematically. Furthermore, the pressure of antlions on different-sized invertebrates also seems to change independently of larval size, increasing either in warmer microhabitats or during the warmer parts of a season. Indeed, Klokočovnik et al. (2016) showed that the capture and consumption of prey by antlion larvae were faster at higher temperatures. Given that antlion larvae can rapidly re-establish their traps, even a brief change in environmental temperatures occurring over days or weeks may significantly alter the pressure of antlion larvae on prey of different sizes. Indeed, Rotkopf et al. (2012) demonstrated that the brief exposure of *M. hyalinus* and *C. lineosa* larvae to elevated temperatures resulted in the enlargement of traps and ultimately increased the speed of prey capture (although the authors did not directly investigate the effects of trap enlargement on the ability to capture larger prey). Overall, we conclude that measurement of the ecological impacts of antlion larvae on natural communities should not ignore their hunting capacities that strongly depend on the thermal environment and larval size. We also emphasize that the thermal ranges used in future experiments addressing the thermal biology of antlion behaviors should match the realistic tolerance limits of the studied species.
